# Effects of handheld nonthermal plasma on the biological responses, mineralization, and inflammatory reactions of polyaryletherketone implant materials

**DOI:** 10.1016/j.jds.2024.06.014

**Published:** 2024-06-27

**Authors:** Chien-Fu Tseng, I-Ta Lee, Sheng-Han Wu, Hsin-Ming Chen, Yuichi Mine, Tzu-Yu Peng, Sang-Heng Kok

**Affiliations:** a Graduate Institute of Clinical Dentistry, School of Dentistry, College of Medicine, National Taiwan University, Taipei, Taiwan; b Department of Dentistry, Taoyunan General Hospital, Ministry of Health and Welfare, Taoyuan, Taiwan; c Research Center of Precision Biomedical Implants, College of Oral Medicine, Taipei Medical University, Taipei, Taiwan; d School of Dentistry, College of Oral Medicine, Taipei Medical University, Taipei, Taiwan; e Graduate Institute of Oral Biology, School of Dentistry, College of Medicine, National Taiwan University, Taipei, Taiwan; f Department of Dentistry, National Taiwan University Hospital, College of Medicine, National Taiwan University, Taipei, Taiwan; g Department of Medical Systems Engineering, Graduate School of Biomedical and Health Sciences, Hiroshima University, Hiroshima, Japan; h Project Research Center for Integrating Digital Dentistry, Hiroshima University, Hiroshima, Japan

**Keywords:** Handheld nonthermal plasma, Dental implant, Polyaryletherketone, Cell metabolic activity, Mineralization, Inflammatory reaction

## Abstract

**Background/purpose:**

The handheld nonthermal plasma (HNP) treatment may alter the surface properties, bone metabolism, and inflammatory reactions of polyaryletherketone (PAEK) dental implant materials. This study tested whether the HNP treatment might increase the biocompatibility, surface hydrophilicity, surface free energies (SFEs), and the cell adhesion and mineralization capability of PAEK materials.

**Materials and methods:**

Disk-shaped samples of titanium (Ti), zirconia (Zr), polyetheretherketone (PEEK [PE]), and polyetherketoneketone (PEKK [PK]) were subjected to HNP treatment and termed as TiPL, ZrPL, PEPL, and PKPL, respectively. Water-surface reactions were examined using a goniometer. MG-63 cells were cultured on all samples to assess the cell viability, cytotoxicity, cell attachment, and mineralization characteristics. The expression of pro-inflammatory cytokines (tumor necrosis factor-alpha and interleukin-6) and key mineralization markers (alkaline phosphatase [ALKP], osteopontin [OPN], and dentin matrix protein 1 [DMP1]) was measured using enzyme-linked immunosorbent assay kits.

**Results:**

The HNP-treated samples exhibited significantly enhanced surface hydrophilicities and SFEs compared to the untreated samples. The cell viability remained high across all samples, indicating no cytotoxic effects. The HNP treatment significantly enhanced MG-63 cell adherence and proliferation. Elevated levels of ALKP and OPN were observed for the plasma-treated PEPL and PKPL specimens, while DMP1 levels increased significantly only in the PKPL specimen. Pro-inflammatory cytokine levels were low across all samples, suggesting no inflammatory response.

**Conclusion:**

The HNP-treated PAEKs have enhanced the surface hydrophilicity and SFEs as well as superior cell adhesion and mineralization capability, and thus may be good clinical dental implant materials.

## Introduction

Dental implants are crucial in the replacement of broken or missing teeth, and their success is closely linked to careful material selection.^1^^,^^2^ Titanium stands as the gold standard material for dental implants due to its biocompatibility, mechanical properties, and microstructure, which promote bone growth.^3^^,^^4^ Yttria-stabilized tetragonal zirconia polycrystals (Y-TZPs) offer an excellent mechanical strength and wear resistance, rendering them particularly suitable for implants bearing heavy loads, such as in extensive loss scenarios or in patients with lower bone densities.^5^^,^^6^ Y-TZP exhibits a superior biocompatibility and integrates well with surrounding tissues, enhancing its implant stability and long-term success rates.^7^^,^^8^ Recently, polymeric materials have gained growing attention in dental implantology. The high-performance semicrystalline thermoplastic polyaryletherketone (PAEK) is known for its outstanding mechanical properties, chemical stability, and biocompatibility. Its molecular structure, containing an aromatic ether and ketone groups, imparts an excellent thermal stability and mechanical performance.^9^^,^^10^ Additionally, PAEK exhibits low density and elastic modulus, resembling natural teeth more closely than traditional materials, thereby reducing stress mismatches between implants and the surrounding tissues to lower implant failure rates.^11^, ^12^, ^13^ However, the inertness and hydrophobicity of the PAEK surface can lower its affinity to biological tissues, consequently reducing the implant stability.^14^^,^^15^

Plasma is a non-destructive treatment technique that effectively enhances the surface properties of materials, wherein the active species and high-energy particles within the plasma are crucial in modifying material surfaces.^16^^,^^17^ Under the influence of electric fields, high-energy charged particles can break existing chemical bonds and reassemble them into new bonds to alter the surface chemistry of the material.^18^ Simultaneously, these high-energy particles continuously erode the material surface, inducing roughness,^4^ and ultimately enhancing the cell affinity, adhesion, and antibacterial properties of the material.^14^ Additionally, active species such as –OH and high-energy particles in the plasma can remove surface contaminants, reduce the relative content of hydrophobic groups (–CH_2_), increase the relative content of oxygen-containing groups (C=O), and decompose large molecular chains while breaking C–H and C–C bonds to achieve surface cleaning and a greater hydrophilicity.^19^^,^^20^ Furthermore, plasma can be utilized for surface grafting and polymerization, generating new activation groups to form strong chemical bonds with other active species.^21^^,^^22^

Many plasma devices used in the semiconductor industry are impractical for dental implant applications due to the requirement for continuous movement of the implants to ensure uniform surface treatment, and the necessity for immediate surgery to avoid atmospheric and functional group interference.^2^ For example, chamber-type plasma treatment requires placing implants on holders within a chamber, wherein they undergo plasma treatment. This treatment approach leads to an improved osseointegration and reduced vertical bone loss, which can shorten the healing time and enhance the implant stability.^4^^,^^19^ Jet-type plasma treatments, on the other hand, are more convenient, utilizing handheld devices to generate highly efficient nonthermal plasma for the surface modification of implants, rendering them more suitable for dental practice.^20^^,^^23^^,^^24^ However, the changes in interactions between water and the implant surface after treatment with handheld nonthermal plasma (HNP) remain unclear. Additionally, further research is necessary to determine whether the inert surfaces of materials such as PAEK can be improved to promote bone cell attachment and metabolism, and to reduce inflammatory reactions. Thus, in the current study, four implant materials were evaluated, namely titanium, zirconia, and emerging polymer materials from the PAEK family (i.e., polyetheretherketone [PEEK] and polyetherketoneketone [PEKK]). The goal was to investigate the characteristic changes in the material surfaces and biological responses after HNP treatment.

## Materials and methods

### Fabrication and surface treatment of the test samples

The materials used in this study and their corresponding abbreviations are listed in Table 1. Disk-shaped samples (ø10.0 mm, thickness 2.5 mm) were fabricated from ASTM grade 5 titanium (Ti), zirconia ceramic (Zr), PEEK (PE), and PEKK (PK) using a dental CAD/CAM milling machine (Milling Unit M1, Zirkonzahn GmbH, Gais, Italy). All samples were subjected to grinding with silicon carbide paper, cleaning with distilled water and isopropyl alcohol in an ultrasonic cleaner, and air-drying. Two surface treatments were applied, namely grinding only and the HNP treatment for 30 s (PiezoBrush PZ3, Relyon Plasma GmbH, Regensburg, Germany). In this study, for clarity, the HNP-treated Ti, Zr, PE, and PK samples were termed as TiPL, ZrPL, PEPL, and PKPL samples, respectively.

**Table 1:** List of materials used.

Trade name (abbreviation)	Main composition^a^	Manufacturer
Coil (Ti)	Ti, Al, V	S-Tech Corp. Tainan City, Taiwan
Superfect Zir (Zr)	ZrO_2_, Y_2_O_3_	Aidite Technology Co., Ltd., Qin Huang Dao, China
BreCAM bioHPP (BP)	PEEK, nanoceramic filler	Bredent, GmbH, Senden, Germany
Pekkton ivory (PK)	PEKK, titanium dioxide	Cendres + Métaux SA, Biel/Bienne, Switzerland

a According to information provided by manufacturers. PEEK: polyetheretherketone; PEKK: polyetherketoneketone.

### Water–surface interactions

The interactions between water and the sample surfaces were evaluated using a goniometer (Phoenix Mini, Surface Electro Optics Co., Ltd., Kunpo, South Korea) to assess their hydrophilic and hydrophobic properties (*n* = 10). In addition to distilled water, this test also included diiodomethane. The Owens-Wendt-Rabel-Kaelble method was employed, utilizing the measured contact angles obtained through the Surfaceware program (v9, Surface Electro Optics Co.) to calculate the surface free energy (SFE).

### Cell cultures

The human osteoblast-like MG-63 cell line (Biosource Collection and Research Center, Hsinchu, Taiwan) was seeded and cultured in minimum essential medium (MEM; Invitrogen, Carlsbad, CA, USA) supplemented with 10% fetal bovine serum, 100 pg/mL streptomycin, and 100 U/mL penicillin. The cells were maintained at 37 °C in a humidified atmosphere containing 5% CO_2_. The culture medium was refreshed every 3 d.

### Cell viability

Autoclaved samples (121 °C, 1.2 kg/cm^2^, 30 min) were placed into a 24-well plate, and the MG-63 cell line was seeded onto each sample at a density of 3 × 10⁶ cells/well. The MG-63 cells were incubated directly with the samples for 24 h and 72 h. Subsequently, the cell viability was assessed using the PrestoBlue cell viability reagent (Invitrogen) in accordance with the manufacturer's instructions. All experimental groups were compared to the Ti group.

### Cell cytotoxicity

Samples of each material were prepared by immersion in the MEM at 37 °C for 72 h. MG-63 cells were seeded onto a 96-well plate at a density of 1 × 10⁶ cells/well. Following cell attachment, the cell culture medium was replaced with the MEM that had been used to immerse each material for 72 h. After 24 h of incubation at 37 °C, the cytotoxicity was evaluated using the PrestoBlue cell viability reagent according to the manufacturer's instructions. All experimental groups were compared to the Ti group.

### Cell attachment and morphology

Samples were seeded with MG-63 for 4 h following the protocols described above. All testing samples were then cleaned with the phosphate-buffered saline, fixed with 4% formaldehyde, dehydrated with alcohol, and dried. The MG-63 cells were attached to each testing sample and were observed by thermal field emission scanning electron microscopy (FE-SEM; JEOL JSM-7800F Prime, JEOL Ltd., Tokyo, Japan). Images were recorded at magnifications of 500× and 5000×.

### Pro-inflammatory cytokine expression

Autoclaved samples (121 °C, 1.2 kg/cm^2^, 30 min) were placed into a 24-well plate, and the MG-63 cells were seeded onto each specimen at a density of 3 × 10⁶ cells/well. The MG-63 cells were incubated directly with the specimens for 72 h. Subsequently, the media were collected, and the levels of interleukin-6 (IL-6) and tumor necrosis factor-alpha (TNF-α) were measured using a human IL-6 ELISA kit (Invitrogen) and a human TNF-α enzyme-linked immunosorbent assay (ELISA) kit (Invitrogen), according to the manufacturer's instructions.

### Mineralization assessment

The expression levels of key markers (alkaline phosphatase [ALKP], osteopontin [OPN], and dentin matrix protein 1 [DMP1]) were measured to assess the material's biocompatibility and its ability to promote bone and tooth mineralization. Autoclaved samples (121 °C, 1.2 kg/cm^2^, 30 min) were placed in a 24-well plate, and the MG-63 cells were seeded onto each sample at a density of 3 × 10⁶ cells/well. The MG-63 cells were incubated directly with the samples for 7 d. Subsequently, the media were collected, and the levels of OPN, DMP1, and ALKP were measured using human OPN, DMP1, and ALKP ELISA kits (Invitrogen) according to the manufacturer's instructions.

### Statistical analysis

The presented data were expressed as the mean ± standard deviation (SD). Normality distribution was confirmed *via* the Shapiro–Wilk test, allowing for parametric tests. The biological evaluation assays were carried out in triplicate. Data were compared using one-way analysis of variance (ANOVA), and Tukey's post-hoc honest significant difference test was used for multiple comparisons among different groups. Statistical analyses were performed using SPSS (v19; IBM Corp., Armonk, NY, USA) and Prism (v10; GraphPad Software Inc., Boston, MA, USA), with the significance set at 5%.

## Results

### Surface characterization

**Figure 1:**
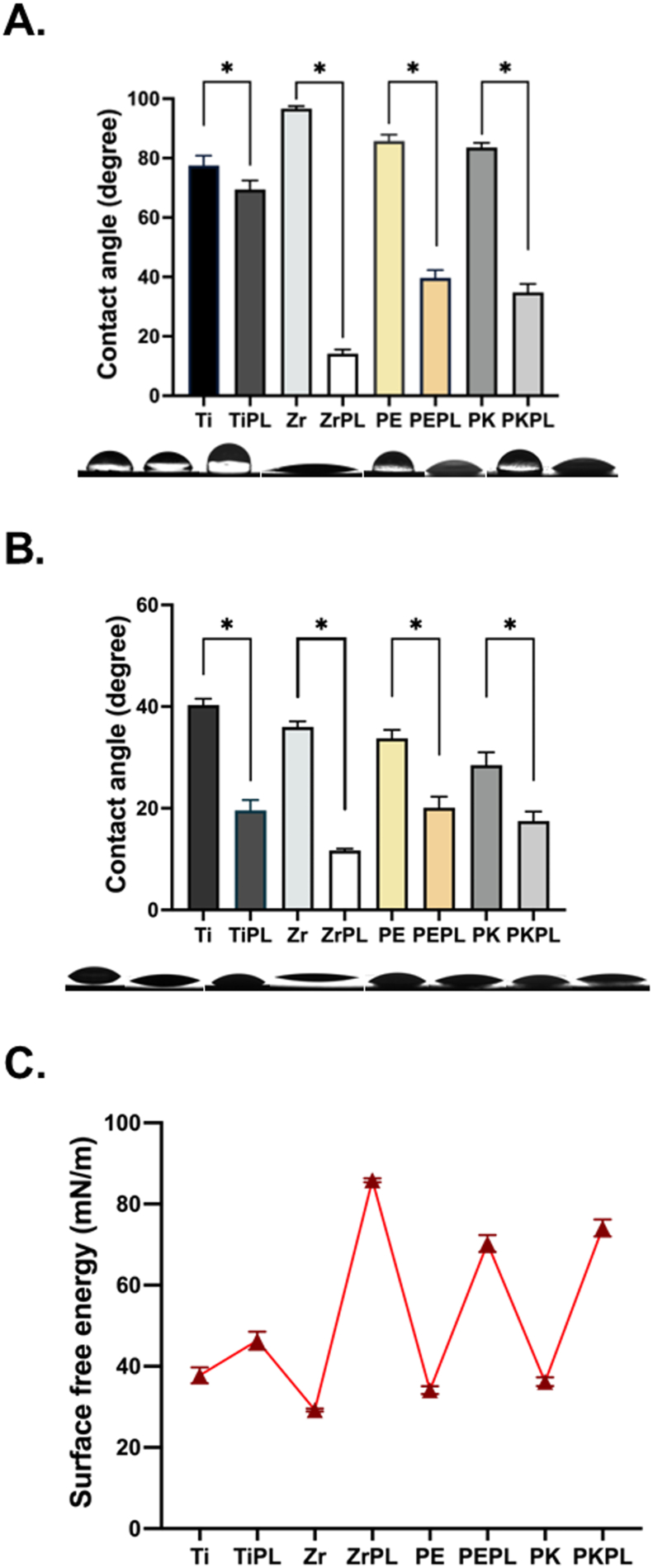
Water–surface reactions of various samples before and after the HNP treatment. The wettability results were determined using (A) distilled water, (B) diiodomethane, and (C) the calculated surface free energy results. For clarity, titanium, zirconia, PEEK, and PEKK samples were designated as Ti, Zr, PE, and PK, respectively, and the HNP-treated samples were termed as TiPL, ZrPL, PEPL, and PKPL, respectively.

The results of the water–surface interaction experiments are presented in Fig. 1 and Table 2. Using distilled water before the HNP treatment, Zr exhibited the most hydrophobic behavior (contact angle = 96.69°), while Ti was the most hydrophilic (77.55°). Interestingly, after the HNP treatment, ZrPL became the most hydrophilic (14.19°), whereas TiPL displayed a hydrophobic surface (69.42°). Using the non-polar solvent diiodomethane, the contact angles were low before plasma treatment, with PK showing a particularly low value. However, after HNP treatment, ZrPL exhibited the lowest contact angle (11.69°). Regardless of the solvent used, the contact angle decreased significantly (*P* < 0.05) after HNP treatment. According to the SFE results, ZrPL had the highest SFE, followed by PKPL and PEPL, thereby indicating that plasma treatment significantly enhanced the SFE (*P* < 0.05).

**Table 2:** The result of contact angle (CA) and surface free energy (SFE).

Groups	CA (degree)		SFE (mN/m)
	Distilled water	Diiodomethane	
Ti	77.55 ± 3.25^a^	40.30 ± 1.26^a^	37.77 ± 1.96^a^
TiPL	69.42 ± 3.03^b^	19.59 ± 2.04^b^	46.31 ± 2.24^b^
Zr	96.69 ± 0.80^a^	35.99 ± 1.10^a^	29.23 ± 0.36^a^
ZrPL	14.19 ± 1.37^b^	11.69 ± 0.37^b^	85.84 ± 0.50^b^
PE	85.75 ± 2.14^a^	33.76 ± 1.65^a^	34.14 ± 0.97^a^
PEPL	39.66 ± 2.66^b^	20.11 ± 2.17^b^	70.26 ± 2,07^b^
PK	83.55 ± 1.56^a^	28.47 ± 2.54^a^	36.21 ± 1.07^a^
PKPL	34.79 ± 2.82^b^	17.51 ± 1.83^b^	74.10 ± 2.09^b^

All the values were presented as mean ± standard deviation.

Within the same column, different letters indicated statistically different groups (*P* < 0.05).

Grouping identification: titanium, zirconia, PEEK, and PEKK were designated as Ti, Zr, PE, and PK, respectively; meanwhile, the HNP-treated materials were termed as TiPL, ZrPL, PEPL, and PKPL, respectively.

### Cell metabolic activity and cytotoxicity

**Figure 2:**
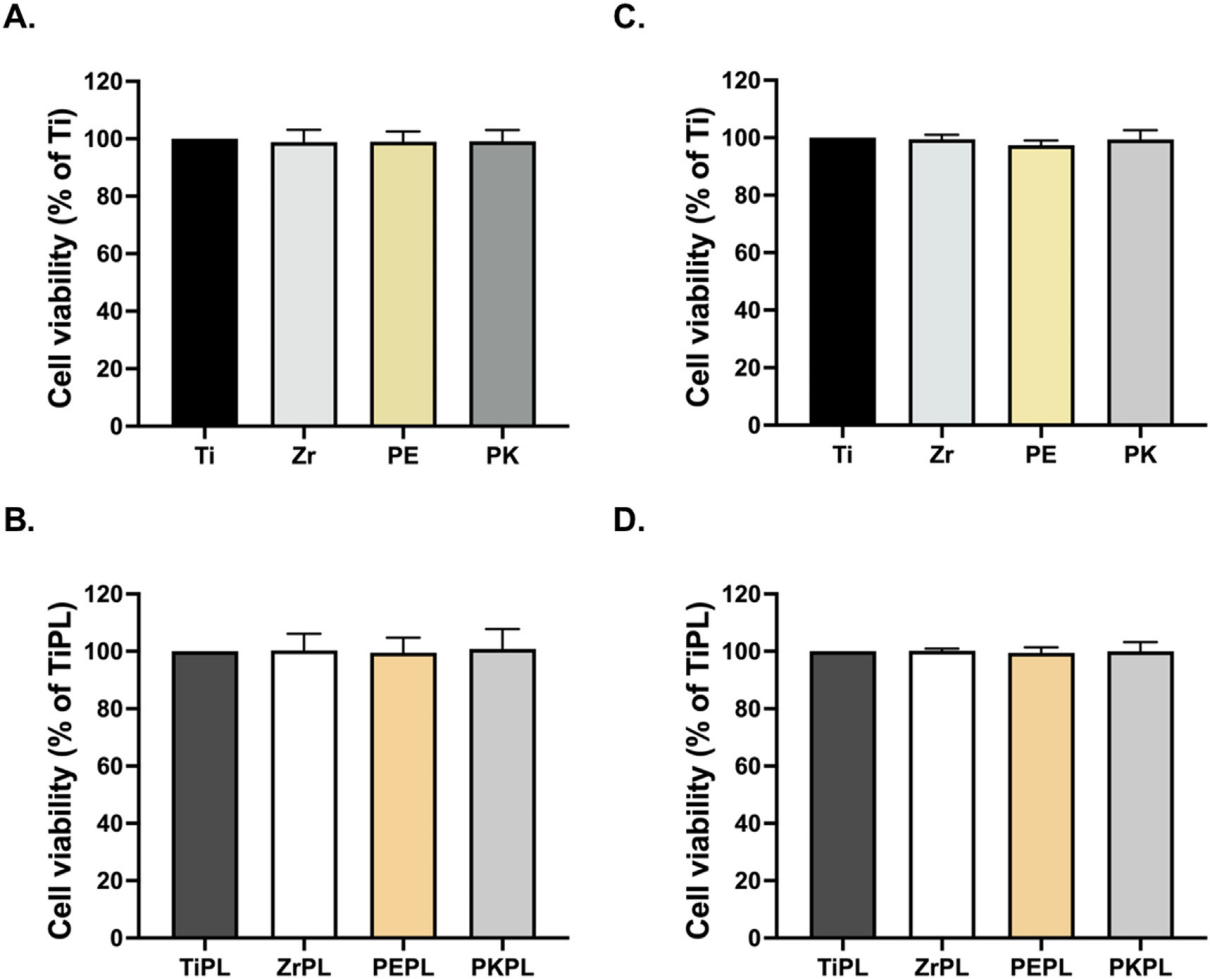
The cell metabolic activity of the MG-63 cells on the various samples. Assessment of the MG-63 cell viability on the samples surface after (A, B) 24 h and (C, D) 72 h, as determined using the PrestoBlue cell viability reagent. For clarity, titanium, zirconia, PEEK, and PEKK samples were designated as Ti, Zr, PE, and PK, respectively, and the HNP-treated samples were termed as TiPL, ZrPL, PEPL, and PKPL, respectively.

**Figure 3:**
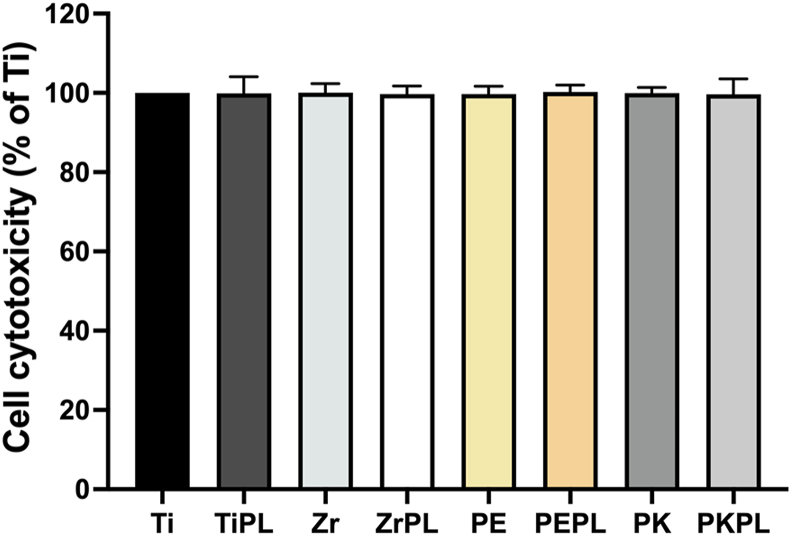
Cytotoxicity assessments of the various samples with relation to the MG-63 cells. The samples were immersed in the MEM at 37 °C for 72 h. After attachment of the MG-63 cells to a 96-well plate, the medium was replaced with the MEM that had been used to immerse the samples for 72 h. The cytotoxicity was evaluated after 24 h using the PrestoBlue cell viability reagent. For clarity, titanium, zirconia, PEEK, and PEKK samples were designated as Ti, Zr, PE, and PK, respectively, and the HNP-treated samples were termed as TiPL, ZrPL, PEPL, and PKPL, respectively.

The cell metabolic activity results are shown in Fig. 2, demonstrating that the viability of MG-63 cells remained high across all material surfaces, regardless of the material type or incubation duration. After 24 h of incubation, the MG-63 cells remained viable on all surfaces, with no significant differences being observed between non-treated or HNP-treated samples (Fig. 2A and B). Similarly, after 72 h, the MG-63 cell viability remained robust across all samples, with the type of material or surface treatment having no significant effect (Fig. 2C and D). The results of the cytotoxicity assessment (Fig. 3) demonstrated that none of the tested samples exhibited a significant cytotoxicity toward the MG-63 cells, with no significant differences being observed after HNP treatment.

### MG-63 cell adherence and morphology

**Figure 4:**
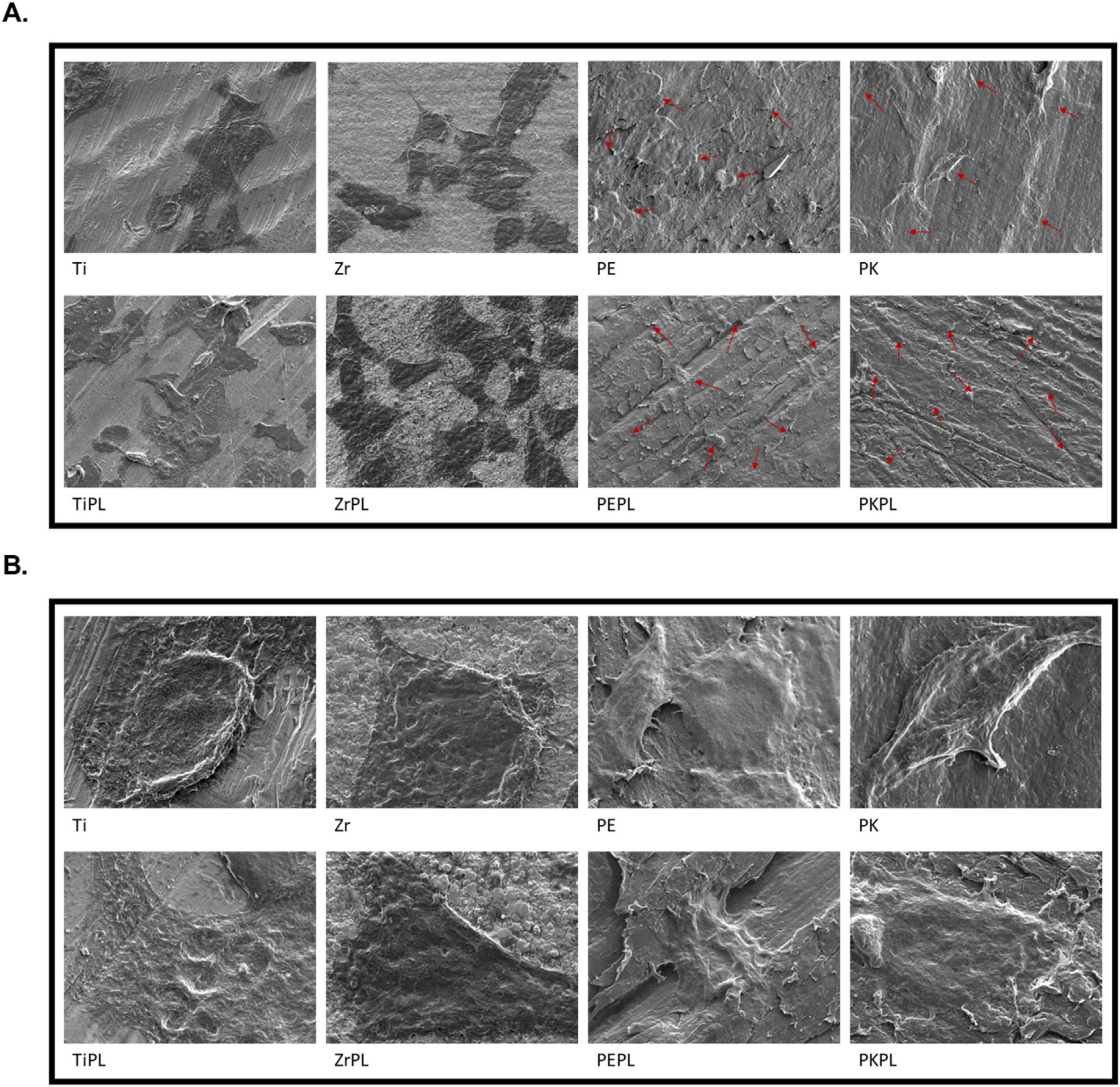
The MG-63 cell adherence characteristics on the sample surfaces. The FE-SEM micrographs obtained after 4 h showing the MG-63 cells adhering to the surfaces of the different materials at magnifications of (A) 500× and (B) 5000×. For clarity, titanium, zirconia, PEEK, and PEKK samples were designated as Ti, Zr, PE, and PK, respectively, and the HNP-treated samples were termed as TiPL, ZrPL, PEPL, and PKPL, respectively. The arrows in the PE, PK, PEPL, and PKPL images indicate the MG-63 cells.

The FE-SEM images obtained after incubation for 4 h revealed the presence of the MG-63 cells (Fig. 4), which adhered to all test samples. Importantly, it was observed that after HNP treatment, the TiPL, ZrPL, PEPL, and PKPL samples exhibited significantly improved cell adherence, suggesting that HNP treatment improves the surface affinity, making it more favorable for the MG-63 cell adhesion and proliferation.

### Pro-inflammatory cytokine expression

**Figure 5:**
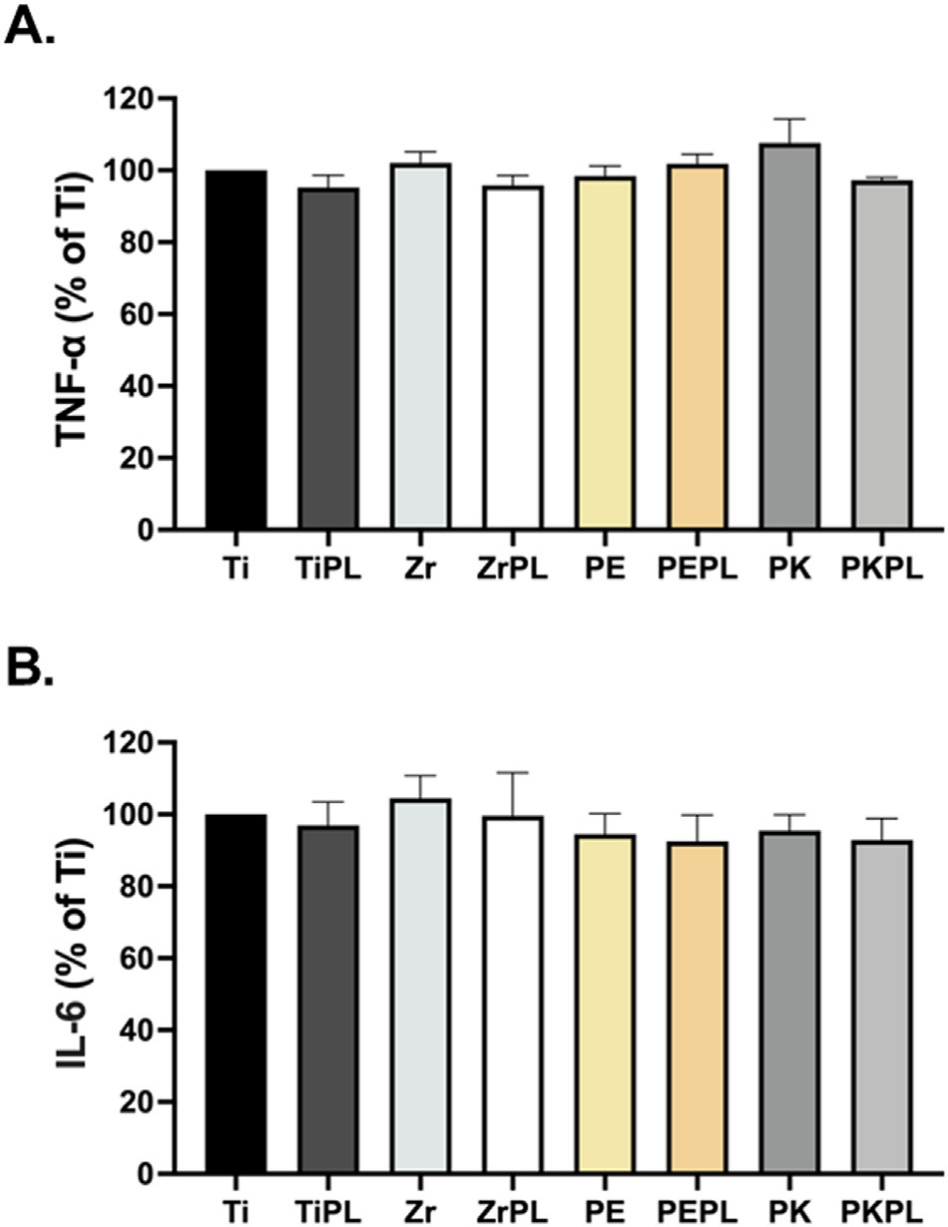
Measurement of pro-inflammatory cytokine expression levels in the MG-63 cells. Autoclaved samples were placed in a 24-well plate, and the MG-63 cells were seeded onto each sample. After 72 h of incubation, the media were collected, and the levels of TNF-α (A) and IL-6 (B) were measured using human TNF-α and IL-6 ELISA kits. For clarity, titanium, zirconia, PEEK, and PEKK samples were designated as Ti, Zr, PE, and PK, respectively, and the HNP-treated samples were termed as TiPL, ZrPL, PEPL, and PKPL, respectively.

The inflammatory reaction results (Fig. 5) demonstrated that the TNF-α and IL-6 levels were low across all samples, indicating that none of the tested samples caused a significant inflammatory response in the MG-63 cells. The induced production of pro-inflammatory cytokines was not observed either before or after the HNP treatment, confirming the biocompatibility and suitability of the HNP-treated specimens for biomedical applications.

### Promotion of bone and tooth mineralization

**Figure 6:**
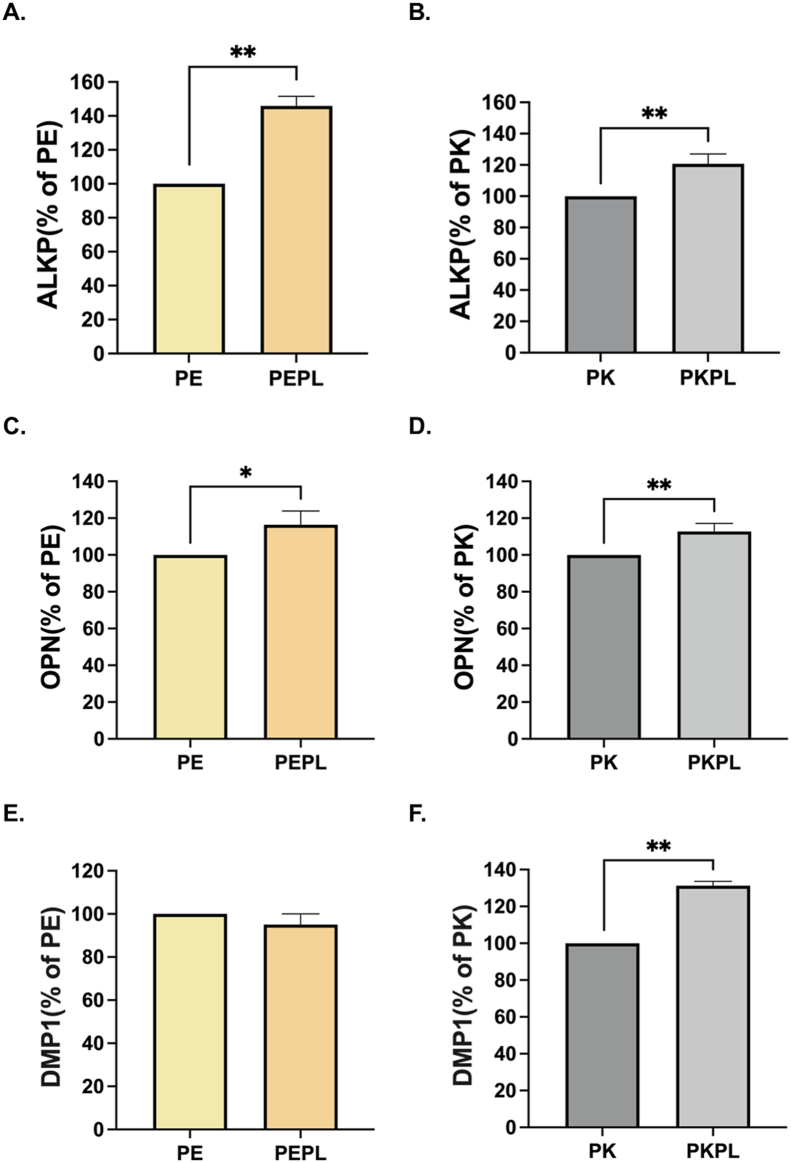
Assessment of the promotion of bone and tooth mineralization in the MG-63 cells incubated with the PE, PEPL, PK, and PKPL. Autoclaved samples were placed in a 24-well plate, and the MG-63 cells were seeded onto each sample. After 7 d of incubation, the media were collected, and the ALKP, OPN, and DMP1 levels were measured. (A, B) ALKP levels, (C, D) OPN levels, (E, F) DMP1 levels. For clarity, titanium, zirconia, PEEK, and PEKK samples were designated as Ti, Zr, PE, and PK, respectively, and the HNP-treated samples were termed as TiPL, ZrPL, PEPL, and PKPL, respectively. ∗*P* < 0.05 and ∗∗*P* < 0.01 represent statistical differences between the two groups.

Subsequently, the biocompatible PAEKs were subjected to bone and tooth mineralization analysis. As shown in Fig. 6, the HNP-treated samples (PEPL and PKPL) significantly increased (*P* < 0.05) the ALKP and OPN expression levels compared to the non-treated samples (PE and PK). However, no significant difference was observed in the DMP1 levels between PE and PEPL, while a significant difference (i.e., an increase) was observed between the PK and PKPL specimens (*P* < 0.05).

## Discussion

The present study aimed to evaluate the effects of HNP treatment on the surface properties, cell viability, cytotoxicity, cell attachment, and mineralization characteristics of PAEK dental implant materials, comparing these results with the traditional dental implant materials. The findings provide significant insights into the potential of using the HNP-treated PAEK materials in dental implantology. One of the most notable outcomes of this study was that the HNP treatment enhanced the surface hydrophilicities and SFEs of the samples (Fig. 1 and Table 2). The dramatic reduction in contact angles, particularly for the ZrPL and PKPL surfaces, aligned with previous studies.^25^^,^^26^ This reduction is critical because it facilitates protein adsorption and subsequent cell adhesion, which are crucial for the successful integration of implants into the surrounding bone tissue. Moreover, an enhanced hydrophilicity and SFE are known to improve the initial interactions between the implant surface and the biological environment, promoting superior osseointegration and potentially reducing the post-implantation healing time.^27^

The high cell viability observed across all dental implant material surfaces indicates an excellent biocompatibility irrespective of the HNP treatment employed (Fig. 2). This is particularly relevant in the context of dental implants, where maintaining a high cell viability is crucial for ensuring the successful osseointegration and long-term implant stability. Previous studies have also shown that plasma treatment does not adversely affect cell survival, further corroborating these findings.^28^ However, plasma treatment had a more pronounced effect on cell adhesion, suggesting that plasma treatment may play a more critical role in enhancing the cell adhesion properties.^29^ In addition, the absence of significant cytotoxicity (Fig. 3) in all tested samples further supports the potential clinical application of these dental implant materials.^30^ This finding is crucial as it underscores the safety profile of the HNP-treated PAEKs, rendering them viable alternatives to the traditional materials like the titanium and zirconia.

The cell adhesion studies (Fig. 4) revealed that the HNP-treated surfaces significantly improved the MG-63 cell adherence and proliferation compared to the non-treated samples. This enhancement can be attributed to the increased surface hydrophilicity and SFE of the treated samples, which promote interactions between the cell membrane and the material surfaces.^31^ This finding is consistent with the results of other studies showing the similar improvements in cell adhesion and proliferation on the HNP-treated material surfaces.^32^ An enhanced cell adhesion is vital for dental implants as it can improve osseointegration and stability, reducing the risk of dental implant failure.^33^ The future research should explore the specific molecular mechanisms by which the HNP treatment enhances cell adhesion and whether these effects are maintained *in vivo*. Moreover, the low levels of pro-inflammatory cytokines across all tested samples indicate that neither the non-treated nor the HNP-treated samples induce significant inflammatory responses in the MG-63 cells (Fig. 5). This is an important finding, since an inflammatory response can lead to the implant failure. These results are consistent with those of the previous studies showing that plasma-treated material surfaces can reduce inflammatory cytokine expression, thereby enhancing the biocompatibilities of the implant materials.^34^ These findings suggest that the HNP-treated PAEKs can be safely used in clinical applications without provoking adverse immune responses, potentially leading to better dental implant outcomes in the patients. Regarding the mineralization potential (Fig. 6), the increased levels of ALKP and OPN in the HNP-treated samples, particularly PEPL and PKPL, highlight the enhanced osteogenic potentials of these material surfaces. This is crucial for applications in bone and dental tissue regeneration, where promoting new bone formation is essential for the long-term success of implants. The elevated expression of these bone mineralization markers suggests that the modified surfaces better support the differentiation and function of osteoblasts, which are crucial for new bone formation around the dental implants. However, the differential response observed in the DMP1 levels indicates that the effects of the HNP treatment on mineralization markers may vary between different PAEKs, warranting further investigation into the specific mechanisms involved.^35^^,^^36^

While this study provides valuable insights, several limitations and areas for the future researches should be considered. Firstly, *in vivo* studies are required to confirm the *in vitro* findings and evaluate the long-term clinical performances of the HNP-treated PAEK materials in the area of dental implantology. Additionally, the specific molecular pathways by which the HNP treatment enhances cell adhesion and mineralization should be elucidated to develop more targeted and effective surface modification strategies. In conclusion, the HNP treatment enhances the surface affinity, cell adhesion, and mineralization potential of PAEKs without inducing cytotoxicity or inflammation. These enhancements position the HNP-treated PAEKs as promising alternatives for the dental implants, potentially offering improved biocompatibilities and performances compared to the traditional dental implant materials.

## Declaration of competing interest

The authors have no conflicts of interest relevant to this article.
